# Red Jujube Juice Fermented with *Pediococcus pentosaceus* and Its Antioxidant Activity in C2C12 Cells

**DOI:** 10.3390/foods15111926

**Published:** 2026-05-29

**Authors:** Ruoqing Liu, Shengchang Zhou, Mei Gao, Zimeng Li, Jingru Xiao, Aerziguli Abulizi, Mingshan Lv, Liang Wang

**Affiliations:** 1 College of Life Science and Technology, Xinjiang University, Urumqi 830017, China; liuruoqing6688@163.com (R.L.); 17591635601@163.com (Z.L.); 2 College of Smart Agriculture (Research Institute), Xinjiang University, Urumqi 830017, China; zhoushengchangsd@163.com (S.Z.); 18119235543@163.com (M.G.);

**Keywords:** red jujube, fermentation, *Pediococcus pentosaceus*, antioxidant activity, sensory quality

## Abstract

As a fruit with both medicinal and edible value, red jujube (*Ziziphus jujuba* Mill.) is abundant in polyphenols and other bioactive components, exhibiting potent antioxidant potential. However, fresh jujubes are highly susceptible to spoilage, while existing processed products suffer from low added value and severe homogenization, restricting the upgrading of the jujube deep-processing industry. In this study, a strain suitable for jujube juice fermentation was selected from six lactic acid bacteria (LAB) strains. Using single-factor tests and response surface methodology, the fermentation conditions of fermented red jujube juice (FRJJ) were optimized. Systematic analysis was performed on the dynamic evolution of physicochemical properties, color, flavor, and bioactive compounds throughout the fermentation process. Additionally, a C2C12 cell model subjected to H_2_O_2_-induced oxidative stress was employed to evaluate the cytoprotective effects and antioxidant activity of FRJJ. The results demonstrated that LAB fermentation increased superoxide dismutase activity and total phenolic content, while improving flavor and sensory quality. Furthermore, FRJJ effectively mitigated oxidative stress damage and enhanced cell viability by reducing intracellular reactive oxygen species levels and maintaining mitochondrial membrane potential. These findings offer a theoretical basis for the value-added utilization of jujube resources and the formulation of functional fermented beverages.

## 1. Introduction

Red jujube (*Ziziphus jujuba* Mill.) known as a medicinal and edible fruit in China, has been cultivated for centuries across extensive planting areas. Chinese major production bases have been established in Xinjiang, Shandong, Shanxi, Hebei, and other provinces, with the industry scale and annual output ranking first in the world [1]. Xinjiang, benefiting from its unique temperate continental climate, abundant sunlight, significant diurnal temperature variation, and clean snowmelt irrigation, has become a core high-quality jujube cultivation base, accounting for over 50% of China’s total output and playing a vital role in the characteristic forest economy and rural industrial development [2]. Jujube fruit is rich in various bioactive components, including polysaccharides [3], polyphenols [4], flavonoids [5], organic acids, vitamins, and cyclic adenosine monophosphate [6]. Among these, polyphenols characterized by high levels of rutin, chlorogenic acid, catechin, and caffeic acid [4] are the primary material basis for their antioxidant effects, effectively eliminating free radicals and mitigating oxidative stress-induced damage. In addition to polyphenols, jujube contains abundant ascorbic acid (vitamin C) and provitamin A carotenoids, as well as organic acids such as citric, malic, and tartaric acids, which collectively contribute to its nutritional and functional properties. These diverse bioactive components make jujube an excellent raw material for developing nutritionally fortified functional foods. However, owing to their physiological characteristics and storage conditions, fresh jujubes with high sugar content and tender flesh are highly susceptible to water loss, decay, and microbial infection after harvest [7]. This leads to a short shelf life and limited circulation, impeding the stable year-round supply of jujube products. Current jujube processing remains dominated by low-value-added products such as dried jujubes, preserved jujubes, and jujube juice [8], while deep-processing technologies are relatively insufficient and product homogeneity is prominent. The conversion and utilization efficiency of bioactive components is low, restricting the industry upgrading toward higher value and functionalization. Therefore, developing green deep-processing technologies that enhance the nutritional value of jujube, extend the industrial chain, and increase product added value has become a crucial direction for promoting high-quality development in the jujube industry [9].

Lactic acid bacteria (LAB) fermentation represents an important biotechnology for achieving high-value utilization, quality improvement, and functional enhancement of fruit and vegetable raw materials [10]. Featuring mild processing conditions, safe and controllable production, and low cost, it has found extensive applications in the deep processing of a broad spectrum of fruits and vegetables, including apples, grapes, mulberries [11], sea buckthorn [12], peaches, apricots [13], tomatoes [14], and peppers. In the fermentation of these fruits and vegetables, commonly used LAB strains include *Limosilactobacillus fermentum*, *Lactiplantibacillus plantarum*, *Lacticaseibacillus casei*, *Lacticaseibacillus paracasei*, etc. These LAB strains produce organic acids such as lactic acid via carbohydrate metabolism, thereby reducing the pH of the system, suppressing the proliferation of spoilage and pathogenic microbes, and ultimately prolonging the shelf life of fruit and vegetable juices. Meanwhile, LAB can promote the release and biotransformation of bound polyphenols and other active components [15], thus increasing the total phenolic content (TPC). These polyphenolic compounds can activate the nuclear factor erythroid 2-related factor 2 (Nrf2) signaling pathway, which upregulates the expression of multiple antioxidant enzymes, including superoxide dismutase (SOD), catalase (CAT), and glutathione peroxidase (GSH-Px) [16]. Consequently, LAB fermentation enhances the activity of these critical antioxidant enzymes and significantly improves the antioxidant capacity and nutritional value of the product. Furthermore, LAB fermentation could alleviate problems such as excessive sweetness and dull taste via metabolic transformation [17], optimize flavor profile and sensory properties, and improve product market acceptance. These studies provide theoretical support for the LAB fermentation of jujube juice and offer a mature and feasible technological pathway for the deep processing and high-value utilization of jujube.

The quality improvement of jujube juice induced by LAB fermentation is attributed to the metabolic adaptability of strains to the matrix and the biotransformation of substrate components mediated by bacterial enzyme systems. LAB consume sugars in jujube juice to produce energy via lactic acid fermentation and maintain intracellular pH homeostasis through acid tolerance mechanisms, thereby supporting their stable proliferation and normal metabolic activities under weakly acidic conditions [18]. Once adapted to the jujube juice matrix, LAB secrete hydrolytic enzymes including glycosidases and esterases to degrade aroma precursors such as glycosidic-bound terpenoids and release free volatile flavor compounds, while LAB themselves synthesize characteristic metabolites including alcohols and esters, collectively enriching the overall aroma characteristics [19]. In addition, LAB consume soluble sugars such as glucose and fructose and accumulate lactic acid during fermentation, which adjusts the sugar-acid ratio and reduces the excessive sweetness of jujube juice, forming a more harmonious flavor profile. Moreover, organic acids and extracellular hydrolases produced during fermentation could cleave glycosidic and ester bonds between polyphenols and cell wall polysaccharides [20], promoting the release of bound phenolic acids and flavonoids. This process exposes active phenolic hydroxyl groups, effectively increases TPC and SOD activity, and further enhances the in vitro antioxidant capacity of jujube juice.

To evaluate the biological relevance of this enhanced antioxidant activity, this study employed the C2C12 cell line, a well-established in vitro model derived from murine skeletal muscle myoblasts for studying oxidative stress and cytoprotection. These cells are highly sensitive to oxidative damage induced by exogenous oxidants such as hydrogen peroxide (H_2_O_2_), allowing for the assessment of intracellular reactive oxygen species (ROS) levels and mitochondrial membrane potential (MMP) as reliable readouts of cytoprotective capacity.

Accordingly, the objectives of this study were to screen suitable LAB strains for jujube juice fermentation and optimize fermentation conditions. Beyond characterizing the physicochemical and flavor variations, we specifically investigated the protective effects of FRJJ against oxidative stress injury in C2C12 cells. This study provides a theoretical basis and technical reference for the deep processing of jujube and the development of functional fermented juice products.

## 2. Materials and Methods

### 2.1. Materials

Jujubes were obtained from a local agricultural suppliers in Hotan (Xinjiang, China), and all experimental materials in this study were adopted from the same batch. *Limosilactobacillus reuteri* CICC 6123, *Pediococcus pentosaceus* CICC 21865, *Lactiplantibacillus plantarum* CICC 25125, *Zymomonas mobilis* CICC 10232, *Lacticaseibacillus casei* CICC 6114, and *Limosilactobacillus fermentum* CICC 25124 were provided by the China Center of Industrial Culture Collection (Beijing, China). Pectinase, cellulase and hemicellulase were supplied by Novozymes (China) Biotechnology Co., Ltd. (Tianjin, China). C2C12 cells were purchased from Procell Life Science & Technology Co., Ltd. (Wuhan, China), with product catalog number CL-0044 and RRID: CVCL_0188. DMEM, fetal bovine serum (FBS) and phosphate-buffered saline (PBS) were sourced from Sangon Biotech (Shanghai) Co., Ltd. (Shanghai, China). 0.05% Trypsin-EDTA and dimethyl sulfoxide (DMSO) were obtained from HyClone (Logan, UT, USA). Assay kits for SOD, Cell Counting Kit-8 (CCK-8), ROS and MMP originated from Nanjing Jiancheng Bioengineering Institute (Nanjing, China). Total sugar content (TSC) and TPC assay kits were provided by Beijing Solarbio Science & Technology Co., Ltd. (Beijing, China).

### 2.2. Strain Activation and Enumeration

One milliliter of LAB suspension preserved in an ultra-low temperature freezer was inoculated into 10 mL of sterile de Man, Rogosa and Sharpe (MRS) broth and incubated at 37 °C for 24 h for subsequent use.

For enumeration, the bacterial suspension was diluted 100-fold with sterile PBS. 10 µL of the diluted solution was added to the edge of a sterile hemocytometer counting chamber, allowing capillary action to fill the chamber without bubbles. The bacterial cells are counted under a microscope to determine their density. During jujube juice fermentation, the biomass is measured every 3 h in accordance with the above method.

### 2.3. Preparation of Fermented Red Jujube Juice

Red jujubes were thoroughly rinsed with tap water to remove surface dust and impurities. Distilled water was then added to the cleaned jujubes at a mass ratio of 1:4 (*w*/*w*), followed by pre-cooking at 85 °C for 15 min. A homogenate was then prepared using a blender. Subsequently, 2‰ (*w*/*w*) pectinase, 1‰ (*w*/*w*) cellulase, and 1‰ (*w*/*w*) hemicellulase (all calculated based on the weight of the raw jujubes) were supplemented for enzymatic hydrolysis at 55 °C for 3 h. After hydrolysis reaction, the jujube juice was pasteurized in a constant temperature water bath (HH-6, Changzhou Guohua Instrument Co., Ltd., Changzhou, China) at 85 °C for 20 min and allowed to cool to room temperature. Activated bacterial suspension was inoculated into the jujube juice. The inoculation volume was calculated based on the viable cell concentration of the suspension to achieve an initial bacterial concentration of 5 × 10^6^ CFU/mL in the jujube juice, followed by batch fermentation without agitation at 37 °C for 22 h. For screening of fermentation strains (Section 2.4), each of the six LAB strains was inoculated separately following the same procedure. For subsequent experiments, *Pediococcus pentosaceus* was used as the starter culture.

### 2.4. Screening of Fermentation Strains

Six different bacterial strains were used individually to ferment jujube homogenate. The initial bacterial concentration was 5 × 10^6^ CFU/mL. After fermentation, the fermentation performance of each strain was evaluated by measuring pH, total soluble solids (TSS), SOD activity, TPC and sensory scores according to the methods described in Section 2.8. The strain exhibiting the highest SOD activity and TPC, along with the best sensory quality, was selected as the optimal starter culture for subsequent experiments.

### 2.5. Bacterial Growth Curve Fitting

Referring to the microbial growth curve fitting method described by Ko et al. [21], nonlinear curve fitting was conducted via Origin 2024 software (OriginLab, Northampton, MA, USA). The Gompertz growth model, classified as a Growth/Sigmoidal function, was used to obtain the bacterial growth curve.

### 2.6. Single-Factor Experiments

For the strain with the better fermentative parameters, single-factor experiments with SOD activity and TPC of FRJJ as response indicators were conducted. The factors examined included fermentation temperature (27, 32, 37, 42, 47 °C), fermentation time (18, 20, 22, 24, 26 h), and inoculum concentration (2, 4, 6, 8, 10 × 10^6^ CFU/mL) to optimize the fermentation conditions.

### 2.7. Response Surface Methodology (RSM) Optimization

Following single-factor optimization, a three-level Box–Behnken design was implemented. Each factor was tested at three distinct levels (Table 1), and the experimental matrix incorporated five replicates at the center point, yielding a total of 17 experimental runs.

### 2.8. Determination of Physicochemical Properties

#### 2.8.1. pH, TSS, and Total Acidity

The pH value of samples was detected by a digital pH meter (PHS-3C, Leici Instrument Co., Ltd., Shanghai, China). A digital refractometer (TD-45, Top Instrument Co., Ltd., Hangzhou, China) was used to test the TSS content. Total acidity was quantified in accordance with the Chinese national standard GB 12456-2021 [22]. All assays were performed in triplicate, and final data were presented as mean ± standard deviation.

#### 2.8.2. SOD, Total Phenolics, and Total Sugars

SOD activity was determined using a total superoxide dismutase (T-SOD) assay kit (hydroxylamine method, A001-1-2, Nanjing Jiancheng Bioengineering Institute, Nanjing, China) according to the manufacturer’s instructions. The absorbance was measured at 550 nm using a microplate reader (Epoch, BioTek, Winooski, VT, USA).

TPC was measured using a plant total phenol assay kit (Folin–Ciocalteu method, BC1345, Beijing Solarbio Science & Technology Co., Ltd., Beijing, China). The absorbance was read at 760 nm using the same microplate reader.

TSC was determined using a total sugar assay kit (DNS method, BC2715, Beijing Solarbio Science & Technology Co., Ltd., Beijing, China). Total sugar was hydrolyzed to reducing sugars by acid, and the reducing sugars reacted with DNS reagent under alkaline heating conditions to form a reddish-brown compound. The absorbance was measured at 540 nm.

#### 2.8.3. Color Difference, Electronic Nose, and Electronic Tongue

Color difference of jujube juice during fermentation was measured using a portable spectrophotometer (DS-700D, Caipu Technology Co., Ltd., Hangzhou, China).

An electronic nose device (PEN3, AIRSENSE Analytics GmbH, Schwerin, Germany) was utilized to characterize the odor profiles of FRJJ samples. In short, 20 mL of the fermentation broth was placed into a sealed vial and maintained at ambient temperature for 30 min to stabilize volatile components before testing. The main instrumental parameters were configured as below: 120 s for measurement and cleaning, 5 s of pre-injection and reset duration, with the carrier gas flow rate fixed at 200 mL/min. Each sample test was repeated three times, and all obtained data were expressed as average values.

A professional taste sensing system (SA402B, Intelligent Sensor Technology, Inc., Atsugi, Japan) was adopted to evaluate nine taste-related indicators, covering sweetness, sourness, saltiness, umami, bitterness, astringency, richness, bitter aftertaste, and astringent aftertaste. Approximately 35 mL of jujube samples collected at various fermentation stages was poured into the sample pool. After the pre-activated sensors completed automatic self-calibration, taste detection was implemented in compliance with standard operating procedures. Meanwhile, the reference solution was set as the blank control to support subsequent data analysis.

#### 2.8.4. Sensory Evaluation

Sensory assessment was carried out referring to the method reported by Liu et al. [23], with slight modifications made in this study. Fifteen trained panelists (balanced male-female ratio, age 25–40 years) tasted randomly coded FRJJ samples. The sensory scoring indices covered appearance, aroma, texture, taste and overall acceptability, with a total score of 100 points. The detailed evaluation criteria are detailed in Table 2. Informed consent was obtained from all panelists prior to participation.

### 2.9. Cell Experiments

#### 2.9.1. Cell Viability

The CCK-8 assay was employed to assess the impacts of RJJ and FRJJ on the viability of C2C12 myoblasts. Briefly, C2C12 cells were plated into 96-well culture plates at a seeding density of 6 × 10^5^ cells per well. When the cell confluence reached 70–80%, the cells were treated with red jujube juice (RJJ) or FRJJ at gradient concentrations (0, 1.5625, 3.125, 6.25, 12.5, 25, and 50 mg/mL) and incubated for 24 h. Following the treatment period, the culture medium in each well was discarded, and 100 µL of DMEM supplemented with 10 µL of CCK-8 reagent was added to each well. After incubation for another 2 h, the absorbance at 450 nm was detected using a multi-mode microplate reader (Synergy H4 Hybrid, BioTek, Winooski, VT, USA). Six replicate wells were prepared for both control and experimental groups, while the blank group contained only culture medium. The cell viability was calculated using the following formula:

Cell viability (%) = (A_sample_ − A_blank_)/(A_control_ − A_blank_) × 100% (1)

where:

A_sample_ is the OD of wells with RJJ/FRJJ-treated cells and CCK-8;

A_blank_ is the OD of wells with medium and CCK-8;

A_control_ is the OD of wells with untreated cells and CCK-8.

#### 2.9.2. Cell Culture and Treatment

C2C12 cells were cultured at 37 °C in a 5% CO_2_ atmosphere with 95% relative humidity. The medium was replaced daily.

To identify the optimal H_2_O_2_ treatment conditions for establishing the cellular oxidative stress model, C2C12 cells were exposed to various concentrations of H_2_O_2_ (0, 125, 250, 500, 1000, and 2000 µmol/L) for 12, 24, and 48 h respectively. According to the obtained cell viability results, the treatment condition of 500 µmol/L H_2_O_2_ for 24 h was chosen to construct the cellular oxidative stress model for the following experiments.

To explore the protective effects of RJJ and FRJJ against H_2_O_2_-induced oxidative damage, the cells were divided into eight groups: control group (medium only), H_2_O_2_ group (medium for 24 h + 500 µmol/L H_2_O_2_ for 24 h), RJJ groups (1.5625, 3.125, or 6.25 mg/mL RJJ for 24 h + 500 µmol/L H_2_O_2_ for 24 h), and FRJJ groups (1.5625, 3.125, or 6.25 mg/mL FRJJ for 24 h + 500 µmol/L H_2_O_2_ for 24 h). After treatment, medium was discarded, and cell viability was measured.

#### 2.9.3. Assessment of Antioxidant Activity

Intracellular ROS levels were detected by DCFH-DA staining (BL714A, Biosharp Co., Ltd., Hefei, China). C2C12 cells were plated in 6-well culture plates at a seeding density of 6 × 10^5^ cells/well and incubated for 24 h. After undergoing the treatment protocol described in Section 2.9.2, the cells were rinsed with PBS, detached with trypsin-EDTA solution, and harvested by centrifugation. Afterwards, the collected cells were incubated with 10 μM fluorescent probe DCFH-DA at 37 °C for 30 min in the dark environment, followed by analysis with a flow cytometer (CytoFLEX, Beckman Coulter, Brea, CA, USA).

MMP was detected using the fluorescent probe JC-1 (2 μM). This probe responds to changes in MMP levels: in normal cells with high MMP, JC-1 forms aggregates in the mitochondrial matrix, producing red fluorescence; in early apoptotic cells with decreased MMP, JC-1 remains as monomers, producing green fluorescence [24]. After being incubated at 37 °C for 20 min in a dark environment, the cells were subjected to flow cytometric analysis.

### 2.10. Statistical Analysis

All experiments were conducted in triplicate, with the resulting data expressed as either mean ± standard deviation or mean values. SPSS 26 software (IBM Corp., Armonk, NY, USA) was utilized to analyze significant differences among multiple groups and between two groups. For the comparison of means among multiple groups, one-way analysis of variance (ANOVA) was performed, followed by Duncan’s multiple range test for post hoc comparison. Response surface optimization was carried out using Design-Expert 13 software (Stat-Ease Inc., Minneapolis, MN, USA), and the significance of the regression model for each response variable was assessed via analysis of variance (ANOVA). The significance of differences in cell viability was examined using Prism software (version 8.0.2, GraphPad, San Diego, CA, USA) with independent-samples *t*-tests. Flow cytometry data were processed and analyzed using FlowJo v10.8.1 software (FlowJo Software, Portland, OR, USA), while all experimental graphs were constructed using Origin 2024 software (OriginLab, Northampton, MA, USA).

## 3. Results and Discussion

### 3.1. Screening of Strains

Different LAB strains display distinct fermentation capabilities in fruit and vegetable matrices. To screen out a strain that has the better values for critical indicators, six LAB strains were individually inoculated for fermentation. Critical indicators, including pH value, TSS, SOD activity, TPC, and sensory evaluation scores of the fermented products, were determined to comprehensively assess the fermentation performance of each strain.

As presented in Table 3, in comparison with the unfermented control group, the FRJJ with different LAB strains significantly decreased its pH value and caused TSS to decline to varying extents (*p* < 0.05). Notably, all six LAB strains were inoculated into red jujube juice prepared under identical conditions, ensuring a comparable initial carbon source baseline. Therefore, the differences among the strains in pH reduction and TSS consumption reflect their varying capacities to utilize soluble sugars for growth and organic acid production. Among all tested strains, *L. plantarum*, *L. reuteri*, and *L. casei* exhibited strong acid-producing capacities, indicating their stronger sugar utilization ability.

As a pivotal antioxidant enzyme, SOD functions to scavenge superoxide anion radicals and safeguard cells against oxidative stress, its activity directly reflects the antioxidant capacity of the system [16]. In this study, jujube juice fermented with *P. pentosaceus* and *L. reuteri* showed significantly higher SOD activity (63.30 U/mL and 62.78 U/mL, respectively) compared to other strains. Previous studies have confirmed that LAB fermentation can increase SOD activity by activating the endogenous antioxidant enzyme system of fruits and vegetables and upregulating the expression of bacterial SOD genes [25,26]. Additionally, reducing substances produced by bacterial metabolism help maintain a reductive microenvironment, which protects the enzyme protein structure from oxidative damage [27]. The combined action of these mechanisms likely explains the significant increase in SOD activity in fermented jujube juice.

Consistent with previous reports that LAB can convert bound polyphenols into free forms through hydrolytic enzymes such as β-glucosidase [28]. TPC significantly increased in all LAB-fermented jujube juice samples in this study. The most significant increases were observed in RJJ fermented with *L. fermentum*, *L. casei*, and *P. pentosaceus* fermentations, reaching 2.02, 1.98, and 1.96 mg/mL, respectively. This further confirms that LAB can effectively mediate the biotransformation of bound polyphenols in jujube and promote the release of free phenolics.

Sensory quality is a core indicator for evaluating the palatability and market acceptance of fermented jujube juice, primarily influenced by the sweet–sour balance, volatile compound composition, and mouthfeel. Compared with unfermented jujube juice, the sensory scores of different LAB strains FRJJ were all improved, with the *P. pentosaceus* group scoring the highest. This is attributed to the organic acids produced during LAB fermentation can effectively regulate the sweet–sour balance of the system, while volatile compounds generated by bacterial metabolism contribute to the unique aroma profile of the juice. Notably, excessive acid production can disrupt flavor balance, resulting in lower sensory scores. Therefore, an ideal fermentation strain should achieve a good balance between acidification for flavor enhancement and overall flavor harmony.

Based on the comprehensive analysis of SOD activity, TPC, and sensory evaluation, *P. pentosaceus* was selected as the optimal strain for fermenting RJJ.

### 3.2. Growth Curve of LAB During FRJJ Fermentation

**Figure 1:**
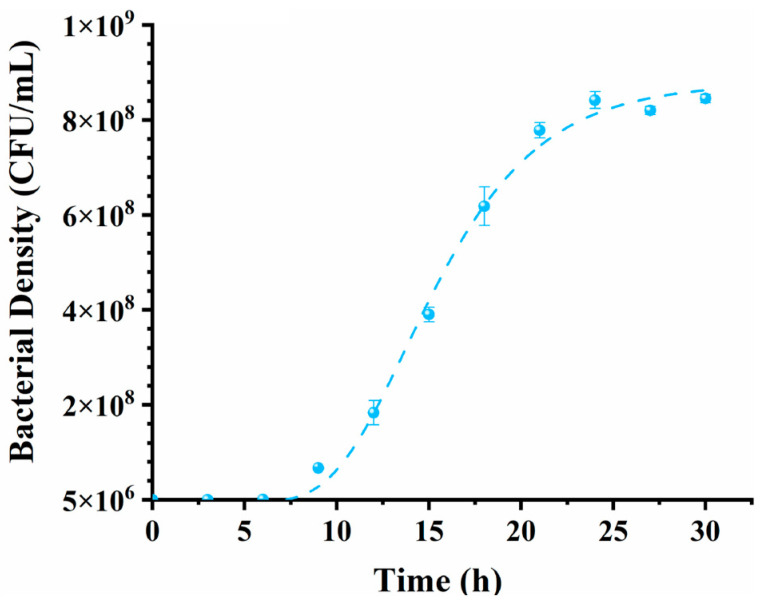
Fitted growth curve of *Pediococcus pentosaceus* in FRJJ. Symbols represent experimental data points, and the line represents the fitted growth curve.

Multiple factors exert an influence on the fermentation of LAB in fruit and vegetable matrices, including substrate adaptability and strain proliferation characteristics [29]. Fitting the growth curve can effectively monitor bacterial proliferation dynamics, thereby determining whether RJJ meets the growth and metabolic requirements of the target strain. Figure 1 shows the fitted growth curve of *P. pentosaceus* in FRJJ, which exhibits a typical “S”-shaped growth pattern, reflecting the complete metabolic process of adaptation, proliferation, and stabilization. During the initial 0–6 h of fermentation, the bacterial density remained low as the strain entered the lag phase. This may be attributed to the time required for the strain to induce sugar transport systems and adjust intracellular osmotic pressure before rapid proliferation [30]. After 6 h, the culture entered the exponential growth phase, with bacterial density increasing rapidly from a low level, showing exponential growth. This indicates that the strain can efficiently utilize soluble sugar, amino acids, and other nutrients in jujube juice, with significantly enhanced metabolic activity, leading to a progressive decrease in pH. The growth curve plateaued around 24 h, coinciding with the depletion of fermentable sugars and the accumulation of acidic metabolites, as the strain entered the stationary phase; the final bacterial density stably maintained above 8 × 10^8^ CFU/mL and no obvious decline phase was observed. These growth characteristics demonstrate that *P. pentosaceus* has excellent adaptability and that jujube juice can fully meet the requirements for its growth, proliferation, and metabolism.

### 3.3. Impacts of Different Fermentation Conditions on Juice Quality

Fermentation temperature, time, and inoculum concentration are critical parameters affecting LAB fermentation. To establish the optimal process conditions for FRJJ, SOD activity and TPC were utilized to carry out the optimization.

**Figure 2:**
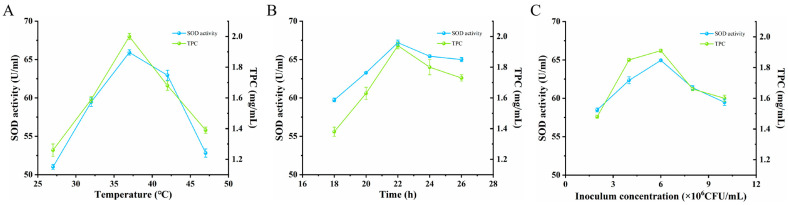
Results of single-factor experiments for FRJJ. (**A**) Fermentation temperature; (**B**) Time; (**C**) Inoculum concentration.

Temperature acts as a dominant environmental factor regulating bacterial growth and metabolism activities, exerting a direct impact on the overall quality and functional characteristics of fermented juice. A suitable temperature facilitates efficient microbial metabolism, whereas excessively high or low temperatures deteriorate fermentation performance. With the fermentation temperature rising from 25 °C to 47 °C, both SOD activity and TPC increased first and then decreased, reaching their maximum values at 37 °C (SOD activity 65.92 U/mL; TPC 2.00 mg/mL). Both indicators declined when the temperature exceeded 37 °C, possibly due to the effects of high temperature on bacterial growth and the oxidative degradation of phenolic compounds (Figure 2A). Consequently, 37 °C was determined to be the optimal fermentation temperature.

As depicted in Figure 2B, SOD activity and TPC increased steadily within 18–22 h and peaked at 22 h (SOD activity 67.20 U/mL; TPC 1.94 mg/mL), followed by a slow decline. This variation trend is highly consistent with the bacterial growth curve. Strains in the exponential growth phase exhibit vigorous metabolic activity, thereby promoting polyphenol biotransformation and SOD accumulation. However, in the stationary phase, nutrient depletion and the accumulation of harmful metabolites can cause a decline in these functional indicators. Hence, 22 h was determined as the optimal fermentation time for subsequent experiments.

Figure 2C demonstrates that at an inoculum concentration of 2 × 10^6^ CFU/mL, the initial bacterial density was insufficient, resulting in slow metabolic initiation and low SOD activity and TPC. Both indicators increased with rising inoculum dosage and reached the highest level at 5 × 10^6^ CFU/mL. When the inoculum concentration exceeded 5 × 10^6^ CFU/mL, excessive LAB proliferation led to intensified nutrient competition, metabolic imbalance, and a rapid drop in pH. Such changes not only compromised the structural stability of SOD but also accelerated the degradation of phenolic compounds. This indicates that a rational inoculum concentration supports rapid strain proliferation and stable metabolism to synthesis functional compounds, whereas an overhigh inoculum breaks the dynamic balance of the fermentation system and weakens functional properties. Consequently, the optimal inoculum concentration was fixed at 5 × 10^6^ CFU/mL.

### 3.4. Optimization of FRJJ by RSM

The single-factor experimental results indicated that fermentation temperature (A), time (B), and inoculum concentration (C) simultaneously influenced SOD activity (Y_1_) and TPC (Y_2_). Following the Box–Behnken design principle, a RSM experiment was carried out. The results of RSM for fermented jujube juice are shown in Table 4.

The corresponding quadratic polynomial regression equations were established as follows:

Y_1_ = 66.04 + 0.9975A + 0.8888B + 0.7887C + 1.77AB − 0.5775AC + 0.755BC − 5.68A^2^ − 2.38B^2^ − 3.42C^2^ (2)

Y_2_ = 1.96 + 0.0388A + 0.0963B + 0.0375C + 0.12AB + 0.0875AC + 0.0725BC − 0.35A^2^ − 0.24B^2^ − 0.0725C^2^ (3)

The ANOVA and significance test results are summarized in Table 5. The fit statistics, including R^2^, Adjusted R^2^, Predicted R^2^, Adeq Precision, and C.V.%, are also listed. The models for both SOD activity and TPC reached high significance with satisfactory fitting degrees, indicating that these models can effectively predict the optimal fermentation conditions. For the SOD activity model, the primary terms (A, B, C), interaction terms (AB, BC), and all quadratic terms were statistically significant, whereas the AC interaction showed no significant difference. All terms in the TPC model were significant. Neither model showed a significant lack of fit, further confirming the reliability of the model fits.

**Figure 3:**
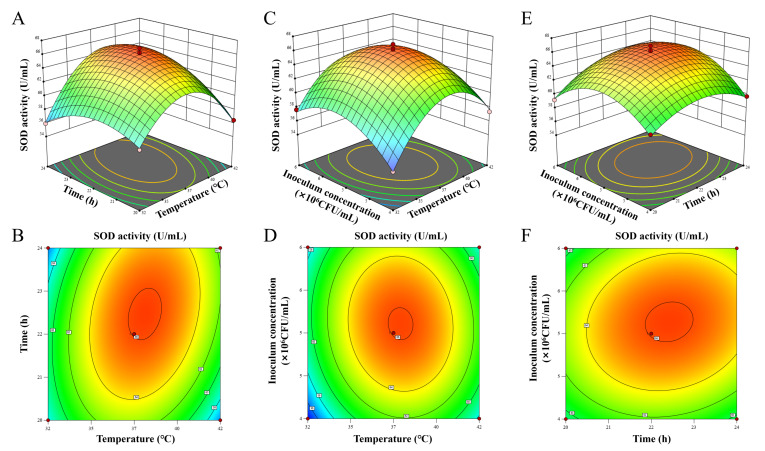
Interaction effects of different factors on SOD. (**A**,**B**) Fermentation time and temperature; (**C**,**D**) Inoculum concentration and temperature; (**E**,**F**) Inoculum concentration and time.

**Figure 4:**
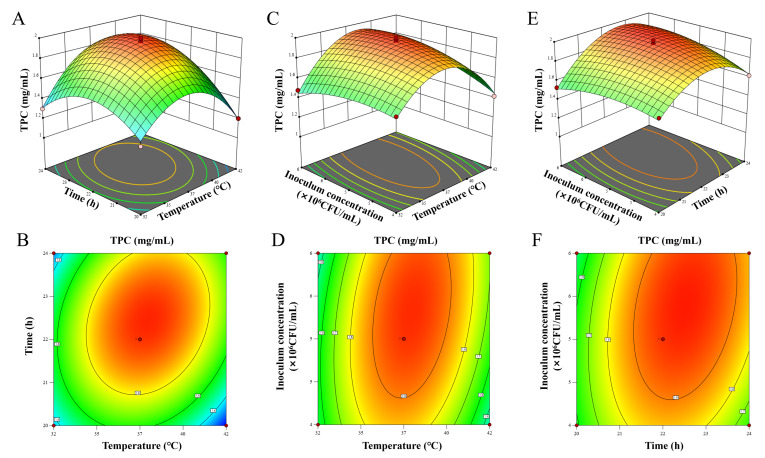
Interaction effects of different factors on TPC. (**A**,**B**) Fermentation time and temperature; (**C**,**D**) Inoculum concentration and temperature; (**E**,**F**) Inoculum concentration and time.

Three-dimensional response surfaces and contour plots were applied to visualize the interactive effects of fermentation temperature, fermentation time and inoculum concentration on SOD activity and TPC (Figure 3 and Figure 4). The steepness of the response surface and the elliptical contour morphology reflect the intensity of factor effects and their interactive relationships [31].

For SOD activity, the response surface corresponding to temperature and time was steep with obviously elliptical contour lines, indicating a highly significant interaction. SOD activity increased initially and then decreased with rising temperature and extended time, reaching a maximum within the optimal range; excessive temperature or over-fermentation resulted in a remarkable reduction in enzyme activity. The response surface between inoculum concentration and temperature was flat with nearly circular contour lines, indicating no significant interaction. Meanwhile, the response plot of inoculum concentration and time verified the significant interaction between these two variables.

Notably, within the tested range (4–6 × 10^6^ CFU/mL), the inoculum concentration had a relatively minor effect on SOD activity and TPC compared to fermentation temperature and time. This indicates that slight variations in inoculum size do not significantly affect the final product quality, demonstrating that the fermentation process is robust to this parameter.

For TPC, all response surfaces exhibited steep slopes with typically elliptical contour lines, showing highly significant interactive effects among variables. TPC likewise increased initially and subsequently decreased as each factor level rose. Under appropriate fermentation conditions, LAB promote the degradation of cell wall structures, facilitating the conversion of bound phenolic into free forms and markedly increasing TPC. The response surfaces of both indicators presented convex and downward-opening parabolic characteristics, confirming the presence of comprehensive optimal values for SOD activity and TPC within the experimental range. These results further supported the feasibility of determining the optimal fermentation parameters via model prediction.

Using the regression model predictions, the optimal fermentation parameters for FRJJ were as follows: fermentation temperature of 37.62 °C, fermentation time of 22.53 h, and LAB inoculum concentration of 5.21 × 10^6^ CFU/mL. Under the aforementioned conditions, the predicted SOD activity and TPC were 66.24 U/mL and 1.98 mg/g. To facilitate practical operation, the parameters were adjusted to 38 °C, 22.5 h, and 5.2 × 10^6^ CFU/mL. Three parallel verification experiments were subsequently performed, the experimental results of SOD activity and TPC were 67.31 U/mL and 1.97 mg/mL, which verified the reliability and practicability of the established regression model.

### 3.5. Changes in Physicochemical Properties During Jujube Juice Fermentation

**Figure 5:**
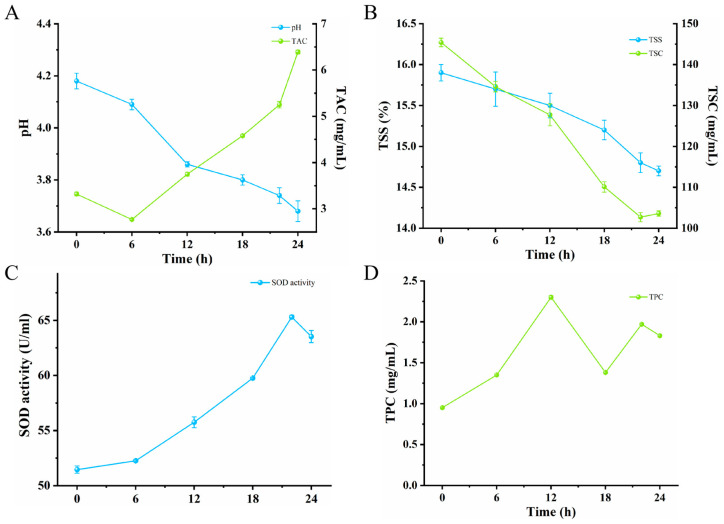
Changes in physicochemical properties of FRJJ during fermentation. (**A**) pH and total acidity; (**B**) total soluble solid and total sugar; (**C**) SOD; (**D**) TPC.

To elucidate the contribution of *P. pentosaceus* during jujube juice fermentation, various physicochemical indicators were measured. As shown in Figure 5A, the pH value continuously decreased from 4.18 to 3.68 within 24 h of fermentation. Total acidity initially decreased slightly from 3.32 to 2.77 mg/mL (0–6 h) and then rapidly increased to 6.39 mg/mL. The pH and total acidity were inversely correlated, primarily because LAB entering the exponential growth phase produce large amounts of organic acids like lactic acid and acetic acid through glycolysis, leading to a continuous increase in acidity. The slight initial decrease in total acidity might be due to the consumption of endogenous organic acids by strains to adapt to the fermentation matrix. This variation is consistent with findings by Mantzourani et al. [32] in mixed fruit juice lactic fermentation, reflecting typical microbial acid production characteristics.

Total sugar serves as the primary carbon source for LAB fermentation; its metabolic variation directly reflects the substrate utilization ability and metabolic intensity of the strain. During jujube juice fermentation, total sugar content decreased significantly from 145.4 mg/mL to 103.6 mg/mL (Figure 5B). The continuous decrease in total sugar concentration was closely associated with the growth curve of *P. pentosaceus*. The total sugar consumption rate was fastest between 12 and 18 h of fermentation, consistent with the exponential growth trend of the strain during this period, indicating that sugar availability is a key parameter controlling growth dynamics and acid production during fermentation. The slight variation in TSS was mainly ascribed to the continuous accumulation of microbial metabolites, such as organic acids and soluble peptides, which partially counterbalanced the reduction in sugar content.

Dynamic variations in SOD activity and TPC were also observed throughout lactic acid fermentation (Figure 5C). SOD activity increased progressively from 51.45 U/mL to 65.29 U/mL and declined slightly at the late fermentation stage. TPC rose rapidly within 0–12 h, and although a moderate reduction occurred from 12 to 18 h, its final value was still considerably higher than that of unfermented jujube juice. The reduction in SOD activity at the later stage may result from continuous pH reduction induced by organic acid accumulation and proteolytic hydrolysis. The overall improvement of TPC was mainly ascribed to the disruption of plant cell wall structures by LAB-derived hydrolases, which promotes the release of bound phenolic compounds, along with the secondary phenolic metabolism of LAB. These findings demonstrate that *P. pentosaceus* can effectively enhance the functional attributes of jujube juice, thereby validating its potential as a promising starter culture for enhancing the antioxidant performance of FRJJ.

### 3.6. Changes in Sensory Properties During Jujube Juice Fermentation

#### 3.6.1. Color Difference Analysis

**Figure 6:**
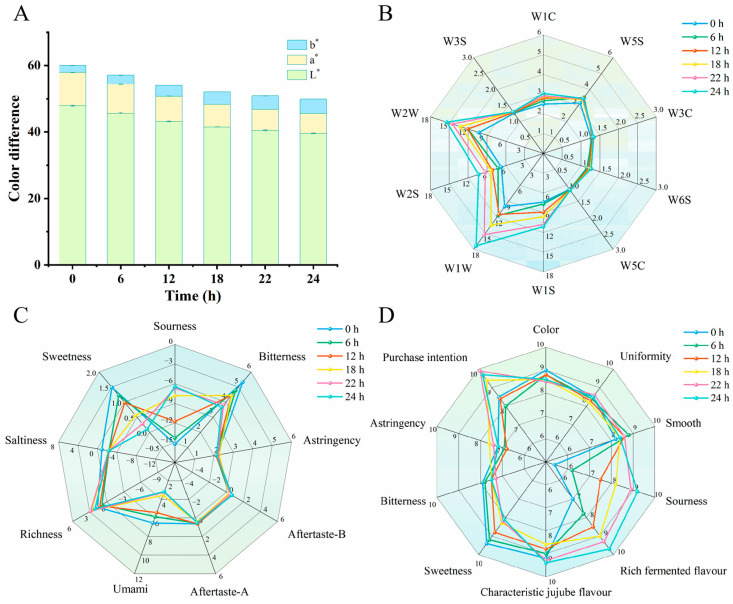
Changes in sensory properties of FRJJ during fermentation. (**A**) Color difference; (**B**) Electronic nose; (**C**) Electronic tongue; (**D**) Sensory evaluation.

Color is a crucial sensory quality index for fermented fruit juices. Color changes during fermentation were measured using a colorimeter (Figure 6A). The lightness value (L*) decreased continuously throughout fermentation, from an initial value of 47.9 to 39.6 at 24 h, indicating that the fermented juice became darker and less bright, transitioning from clear transparency to turbidity. This is closely related to the turbid sediment produced by LAB metabolism and the oxidative polymerization of polyphenols. The redness (a*) and yellowness (b*) values remained relatively stable with slight fluctuations over the fermentation period. The marginal reduction in a* and mild elevation in b* reflects the dynamic transformation of color-related components, including carotenoids, anthocyanins, and flavonoids in jujube matrix. Metabolites produced by *P. pentosaceus* effectively suppress the activity of polyphenol oxidase, restrict pigment degradation, and consequently preserve the color stability of fermented jujube juice. This result is consistent with the findings of Shen et al. [33], who reported that *P. pentosaceus* SSC12 positively regulates anthocyanin metabolism, inhibits browning, and maintains litchi fruit color.

#### 3.6.2. Electronic Nose Analysis

The electronic nose is a bionic instrument that simulates human olfactory perception and can rapidly distinguish diverse volatile flavor compounds in food samples. The radar plot (Figure 6B) revealed obvious differences in electronic nose responses of FRJJ at different fermentation stages. Sensors W1W, W2W, W1S, and W2S presented relatively high response values, suggesting that prolonged fermentation gradually elevated the contents of aromatic substances, inorganic and organic sulfides, methyl compounds, alcohols, aldehydes, and ketones. The increased signals of W1W and W2W were mainly result from the bacterial metabolism. *P. pentosaceus* utilized sugar and amino acid precursors in jujube juice to produce sulfur-containing volatiles, aromatic metabolites, alcohols, and aldehydes, thus enhancing the overall aroma profile. The rising response of W2S further supports this result. In contrast, other sensors (W3S, W1C, W5S, W3C, W6S, W5C) kept low and stable responses, suggesting low accumulation of alkanes, benzene derivatives, nitrogen oxides, ammonia, and hydrides in FRJJ. These results illustrated that LAB fermentation did not produce unpleasant off-flavors, which guaranteed favorable flavor harmony. In summary, *P. pentosaceus* fermentation effectively reshaped the flavor characteristics of jujube juice, converting the original single sweet jujube taste into a fermented product with more abundant and layered aromatic notes.

#### 3.6.3. Electronic Tongue Analysis

Electronic tongue technology was applied to evaluate taste variations. As illustrated in Figure 6C, obvious differences in taste attributes were observed among samples collected at various time points. Sourness increased significantly and continuously throughout fermentation, whereas sweetness gradually decreased. This aligns with the physicochemical results showing LAB metabolizing soluble sugars to produce acids, representing the core aspect of the typical lactic acid flavor imparted to FRJJ by fermentation.

The bitterness response gradually declined during fermentation, which was primarily due to the degradation of bitter glycosides by hydrolytic enzymes secreted by the strain. A similar metabolic mechanism has been well documented in wine fermentation research reported by Wang et al. [34]. Regarding umami, its response value decreased in the early fermentation phase and remained stable after 18 h, which was closely associated with the dynamic balance of flavor amino acids. In the initial stage, the strain preferentially utilized free amino acids to support proliferation. In the middle and late fermentation periods, microbial proteases progressively hydrolyzed macromolecular proteins into small-molecule amino acids. The continuous consumption and de novo generation of umami-related compounds eventually reached a steady state, leading to the stabilization of umami intensity. Additionally, astringency, saltiness, bitter aftertaste, and astringent aftertaste maintained consistently low levels with no remarkable fluctuations during the whole fermentation process. These findings indicated that LAB fermentation effectively avoided undesirable taste defects, thereby guaranteeing satisfactory taste harmony and sensory acceptability of the final product.

#### 3.6.4. Sensory Evaluation Analysis

Sensory evaluation serves as an indispensable indicator for beverage quality assessment, which directly reflects comprehensive flavor attributes and determines consumer acceptability and purchase preference. As shown in Figure 6D, FRJJ collected at different fermentation stages presented a uniform and bright appearance without visible impurities, accompanied by a fine texture and distinct jujube aroma.

Unfermented juice (0 h) presented a typical profile of high sweetness and low sourness. As fermentation progressed, sweetness gradually decreased while sourness significantly increased, consistent with the metabolic process of *P. pentosaceus* consuming sugars and producing organic acids. This effectively alleviated the excessive sweetness of the original juice and achieved an ideal sweet–sour balance during the 18–22 h period. Notably, the scores for “rich fermented flavor” and “characteristic jujube flavor” increased in parallel, indicating that fermentation did not mask the inherent jujube aroma but rather enriched the flavor complexity through the generation of volatile compounds. Throughout the fermentation process, astringency remained at a low level, while “smoothness” scores gradually increased; together, these two attributes ensured a round and harmonious mouthfeel. The overall enhancement of flavor complexity was highly consistent with consumer acceptability trends, confirming the key role of *P. pentosaceus* fermentation in improving the sensory quality and commercial potential of jujube juice.

### 3.7. In Vitro Antioxidant Activity Evaluation of RJJ and FRJJ

#### 3.7.1. Effect of RJJ and FRJJ on C2C12 Cell Viability

**Figure 7:**
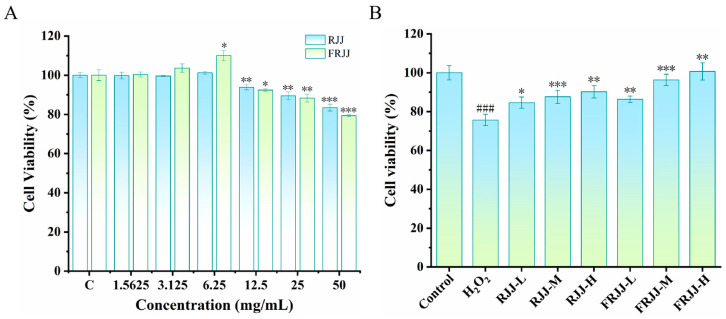
Effect of different treatments on cell viability. (**A**) Effect of RJJ and FRJJ on normal cells (vs. Control group: * *p* < 0.05, ** *p* < 0.01, *** *p* < 0.001); (**B**) Effect of RJJ and FRJJ on H_2_O_2_-treated cells (vs. Control group: ### *p* < 0.001; compared with H_2_O_2_ injury group: * *p* < 0.05, ** *p* < 0.01, *** *p* < 0.001).

To screen the maximum safe and effective concentration of RJJ and FRJJ for normal C2C12 cells, cell viability was determined after 24 h of exposure to gradient sample concentrations (0, 1.5625, 3.125, 6.25, 12.5, 25, 50 mg/mL). As illustrated in Figure 7A, both RJJ and FRJJ exhibited concentration-dependent cytotoxicity relative to the control group. RJJ had no significant toxic effect up to 6.25 mg/mL (*p* > 0.05), and FRJJ up to 3.125 mg/mL (*p* > 0.05). In particular, FRJJ at 6.25 mg/mL markedly facilitated cell proliferation, increasing cell viability by 10.02%. In contrast, concentrations exceeding 12.5 mg/mL significantly inhibited the viability of C2C12 cells in both RJJ and FRJJ groups. Therefore, 6.25 mg/mL was defined as the maximum safe and effective concentration for subsequent cell experiments. To eliminate the interference of intrinsic cytotoxicity, three concentrations (1.5625, 3.125, and 6.25 mg/mL) were selected as low, medium, and high doses for the following oxidative stress protection assays.

#### 3.7.2. Effect of RJJ and FRJJ at Different Doses on the Viability of H_2_O_2_-Stressed C2C12 Cells

Oxidative stress is a core pathological mechanism responsible for cell damage, senescence, and the progression of multiple chronic diseases. As a classic exogenous oxidative stress inducer, H_2_O_2_ triggers excessive ROS accumulation, breaks intracellular redox homeostasis, and ultimately suppresses cell viability [35]. To further compare the cellular antioxidant capacities of RJJ and FRJJ, an oxidative damage model in C2C12 cells was constructed via H_2_O_2_ stimulation.

As presented in Figure 7B, the 24 h H_2_O_2_ treatment reduced cell viability by 24.30% relative to the normal control group (*p* < 0.001), successfully establishing a stable oxidative injury model. Compared with the model group, pretreatment with low, medium, and high doses RJJ and FRJJ distinctly elevated the viability of H_2_O_2_-damaged C2C12 cells. Among these, high-dose FRJJ exerted the most remarkable cytoprotective effect, with cell viability increased by 24.92% relative to the model group, reaching a survival rate approached the level of the normal control group. FRJJ exhibited stronger protection against H_2_O_2_-induced oxidative damage in C2C12 cells, indicating that *P. pentosaceus* fermentation enhanced the antioxidant activity of jujube juice. Mechanistically, the protective effect of FRJJ might involve direct scavenging of intracellular ROS by its bioactive components (e.g., phenolic compounds). Additionally, the near-complete recovery of cell viability after high-dose FRJJ treatment suggests a possible activation of intracellular antioxidant defense systems.

#### 3.7.3. Effect of RJJ and FRJJ on Intracellular ROS Levels

**Figure 8:**
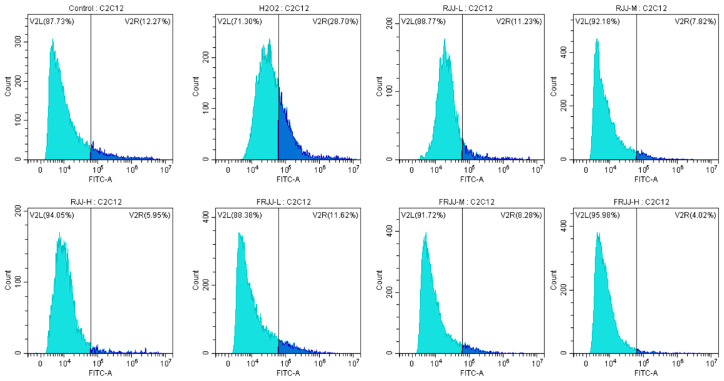
Effect of different doses of RJJ and FRJJ on H_2_O_2_-induced intracellular reactive oxygen species production in C2C12 cells.

Abnormal accumulation of intracellular ROS is a central feature of oxidative stress. The ability to effectively scavenge or inhibit excessive ROS generation is used to evaluate the antioxidant potential of substances [36]. To explore the mechanism by which RJJ and FRJJ alleviate H_2_O_2_-induced oxidative stress, intracellular ROS levels were measured (Figure 8). Compared to the normal group, intracellular ROS levels increased in the H_2_O_2_-treated group, while treatment with RJJ and FRJJ reversed this trend. ROS production in model group increased by 16.43%. In contrast, high-dose RJJ reduced ROS fluorescence intensity by 22.75%, and high-dose FRJJ achieved a reduction of 24.68%, verifying the superior antioxidant performance of FRJJ at the same dosage. Collectively, both RJJ and FRJJ could effectively scavenge H_2_O_2_-induced overproduction of ROS, thereby mitigating cellular oxidative stress damage.

#### 3.7.4. Effect of RJJ and FRJJ on MMP

MMP stability is essential for sustaining normal mitochondrial function and suppressing endogenous apoptosis, and its alterations are tightly associated with cellular oxidative stress [37]. To investigate whether the protective effects of RJJ and FRJJ against oxidative damage are mediated via mitochondrial regulation, MMP in C2C12 cells was detected through JC-1 staining.

**Figure 9:**
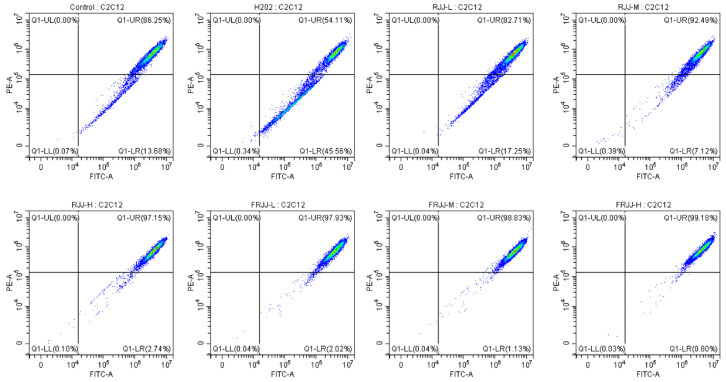
Effect of different doses of RJJ and FRJJ on H_2_O_2_-induced mitochondrial membrane potential in C2C12 cells.

As illustrated in Figure 9, the model group displayed a decline in MMP relative to the control group. The proportion of cells with normal MMP decreased from 86.25% to 54.11%, suggesting that H_2_O_2_ exposure triggered mitochondrial dysfunction and further initiated endogenous apoptosis. However, pretreatment with graded concentrations of RJJ and FRJJ effectively restored the declined MMP. High-dose RJJ recovered the MMP level to 97.15%, while FRJJ presented a more prominent mitochondrial protective capacity; the proportion of cells with intact MMP reached 99.18% in the high-dose FRJJ group. Similar to our findings, another research demonstrated that jujube fruit fermented by gut microbiota could increase cellular bioactivity and antioxidant capacity by reducing intracellular ROS accumulation and restoring MMP [38]. In conclusion, both RJJ and FRJJ can alleviate oxidative stress injury by maintaining MMP stability and protecting mitochondrial structure and function, with the fermentation process further enhancing this protective effect.

## 4. Conclusions

This study confirmed that LAB fermentation can serve as an effective bioprocessing strategy to simultaneously enhance the nutritional and sensory properties of RJJ. Fermentation significantly regulated the sugar-acid ratio, enhanced SOD activity and TPC, and optimized the overall odor and taste profiles. Such modifications relieved the excessive sweetness and single flavor defect of raw jujube juice, thereby greatly improving palatability and commercial application potential. In vitro cell assays verified that both RJJ and FRJJ exerted prominent antioxidant effects by scavenging intracellular ROS and maintaining MMP, ultimately mitigating H_2_O_2_-induced oxidative damage in C2C12 cells. Notably, FRJJ exhibited superior antioxidant activity and mitochondrial stability than RJJ. These findings suggest that FRJJ is more suitable as a functional beverage possessing nutritional value, favorable flavor, and antioxidant activity than its unfermented counterpart. This study provides theoretical and technical support for the application of probiotic fermentation technology in the deep processing of jujube. The established methodology also offers a reference for developing similar fermented fruit and vegetable products.

Future work may focus on identifying and quantifying the phytochemical compounds modified or generated during *P. pentosaceus* fermentation, particularly polyphenols and organic acids. High-performance liquid chromatography could be employed to precisely quantify various sugars and organic acids, and multi-omics approaches could be integrated to systematically dissect the metabolic regulatory network of *P. pentosaceus* during jujube juice fermentation, which would help more comprehensively elucidate its fermentation mechanism and the formation of product quality attributes. Such characterization would clarify the specific bioactive metabolites contributing to the enhanced antioxidant capacity and oxidative stress reduction observed in FRJJ. In addition, in vivo experiments are warranted to systematically assess the bioactivity and safety of FRJJ, providing more comprehensive insights to support its large-scale production and practical utilization.

## Figures and Tables

**Table 1:** Coded and actual levels of independent variables for response surface methodology (RSM).

Factors	Code	Levels		
		−1	0	1
Temperature (°C)	A	32	37	42
Time (h)	B	20	22	24
Inoculum concentration (×10^6^ CFU/mL)	C	4	5	6

**Table 2:** Sensory evaluation criteria for fermented red jujube juice (FRJJ).

Scoring Items	Scoring Criteria	Score
Appearance	Color	0–10
	Uniformity	0–10
Aroma	Characteristic jujube flavour	0–10
	Rich fermented flavour	0–10
Texture	Smooth	0–10
Taste	Sourness	0–10
	Sweetness	0–10
	Bitterness	0–10
	Astringency	0–10
Acceptability	Purchase intention	0–10

**Table 3:** Fermentation performance of different lactic acid bacteria strains on RJJ.

No.	Strain	pH	TSS (%)	SOD (U/mL)	TPC (mg/mL)	Sensory Score
1	Unfermented	4.16 ± 0.02 ^a^	15.7 ± 0.15 ^a^	52.15 ± 0.32 ^e^	0.95 ± 0.01 ^f^	80.27 ± 0.42 ^d^
2	*L. reuteri*	3.48 ± 0.01 ^e^	14.8 ± 0.15 ^c^	62.78 ± 0.29 ^a^	1.79 ± 0.01 ^de^	84.20 ± 0.26 ^b^
3	*P. pentosaceus*	3.73 ± 0.01 ^b^	15.0 ± 0.12 ^bc^	63.30 ± 0.34 ^a^	1.98 ± 0.01 ^b^	86.63 ± 0.91 ^a^
4	*L. plantarum*	3.45 ± 0.02 ^e^	15.1 ± 0.06 ^bc^	59.15 ± 0.57 ^c^	1.77 ± 0.01 ^e^	82.47 ± 0.60 ^c^
5	*Z. mobilis*	3.56 ± 0.02 ^d^	14.9 ± 0.25 ^bc^	58.37 ± 0.31 ^c^	1.80 ± 0.01 ^d^	82.07 ± 0.65 ^c^
6	*L. casei*	3.46 ± 0.01 ^e^	15.0 ± 0.25 ^bc^	61.36 ± 0.35 ^b^	1.96 ± 0.00 ^c^	81.67 ± 1.21 ^cd^
7	*L. fermentum*	3.62 ± 0.03 ^c^	15.2 ± 0.10 ^b^	55.13 ± 0.58 ^d^	2.02 ± 0.02 ^a^	85.27 ± 0.57 ^ab^

Different lowercase letters in the upper right corner of values within the same column denote significant differences among the values (*p* < 0.05).

**Table 4:** Response surface optimisation for FRJJ.

No.	Factors			Response Values	
	Temperature (°C)	Time (h)	Inoculum Concentration (×10^6^ CFU/mL)	SOD Activity (U/mL)	TPC (mg/mL)
1	32	20	5	57.67 ± 0.27	1.31 ± 0.04
2	42	20	5	56.46 ± 0.32	1.20 ± 0.02
3	32	24	5	55.96 ± 0.58	1.30 ± 0.04
4	42	24	5	61.84 ± 0.70	1.67 ± 0.03
5	32	22	4	54.56 ± 0.31	1.57 ± 0.04
6	42	22	4	57.37 ± 0.49	1.42 ± 0.02
7	32	22	6	57.66 ± 0.57	1.48 ± 0.03
8	42	22	6	58.16 ± 0.26	1.68 ± 0.05
9	37	20	4	59.53 ± 0.34	1.61 ± 0.02
10	37	24	4	59.74 ± 0.58	1.62 ± 0.01
11	37	20	6	59.23 ± 0.55	1.53 ± 0.03
12	37	24	6	62.46 ± 0.61	1.83 ± 0.05
13	37	22	5	65.13 ± 0.29	1.97 ± 0.00
14	37	22	5	66.21 ± 0.65	1.96 ± 0.05
15	37	22	5	66.29 ± 0.31	1.92 ± 0.04
16	37	22	5	65.64 ± 0.19	1.95 ± 0.01
17	37	22	5	66.91 ± 0.67	2.00 ± 0.05

**Table 5:** ANOVA for Superoxide dismutase (SOD) activity and total phenolic content (TPC) in FRJJ.

Source	SOD Activity (U/mL)		TPC (mg/mL)	
	F-Value	*p*-Value	F-Value	*p*-Value
Model	87.84	<0.0001	68.57	<0.0001
A-Temperature	23.77	0.0018	7.05	0.0327
B-Time	18.87	0.0034	43.50	0.0003
C-Inoculum concentration	14.86	0.0063	6.60	0.0370
AB	37.53	0.0005	33.81	0.0007
AC	3.98	0.0861	17.98	0.0038
BC	6.81	0.0349	12.34	0.0098
A^2^	405.34	<0.0001	302.77	<0.0001
B^2^	70.95	<0.0001	142.36	<0.0001
C^2^	147.10	<0.0001	12.99	0.0087
Lack of Fit	0.3688	0.7806	3.34	0.1371
R^2^	0.9912		0.9888	
Adjusted R^2^	0.9799		0.9744	
Predicted R^2^	0.9588		0.8667	
Adeq Precision	25.8251		24.2450	
C.V. %	0.9544		2.50	

## Data Availability

The original contributions presented in the study are included in the article; further inquiries can be directed to the corresponding authors.

## Abbreviations

The following abbreviations are used in this manuscript:

LAB	lactic acid bacteria
FRJJ	fermented red jujube juice
RSM	response surface methodology
SOD	superoxide dismutase
ROS	reactive oxygen species
MMP	mitochondrial membrane potential
TPC	total phenolic content
FBS	fetal bovine serum
PBS	phosphate-buffered saline
DMSO	dimethyl sulfoxide
CCK-8	cell counting kit-8
TSC	total sugar content
BBD	Box–Behnken design
TSS	total soluble solid
MRS	De Man, Rogosa and Sharpe
DMEM	Dulbecco’s Modified Eagle Medium
